# Combination inhibition activity of chlorhexidine and antibiotics on multidrug-resistant *Acinetobacter baumannii* in vitro

**DOI:** 10.1186/s12879-021-05963-6

**Published:** 2021-03-17

**Authors:** Fei Lin, Bin Yu, Qinghui Wang, Mingyong Yuan, Baodong Ling

**Affiliations:** 1 Department of Pharmacy, Clinical Medical College and The First Affiliated Hospital of Chengdu Medical College, Chengdu, Sichuan China; 2 Department of Pharmacy, Mianyang Central Hospital, Mianyang, Sichuan China; 3 Sichuan Province College Key Laboratory of Structure-Specific Small Molecule Drugs, School of Pharmacy, Chengdu Medical College, Chengdu, Sichuan China

**Keywords:** *A. baumannii*, Chlorhexidine, Antibiotics, Antiseptics, In vitro

## Abstract

**Background:**

Chlorhexidine is a widely used disinfectant in clinical settings and a broad-spectrum antimicrobial agent effective against aerobic and anaerobic bacteria. However, disinfectant resistant or non-susceptible bacteria, including antibiotic-resistant *Acinetobacter baumannii,* have been found. This study aimed to develop a new technique to prevent and control *A. baumannii* infection in the hospital setting.

**Methods:**

Chlorhexidine combined with minocycline, doxycycline, meropenem, imipenem, levofloxacin and ciprofloxacin were tested against the 30 multidrug-resistant and extremely drug-resistant *A. baumannii* clinical isolates. The checkerboard test was used to calculate the fractional inhibitory concentration index according to the minimum inhibitory concentration value for chlorhexidine combined with antibiotics.

**Results:**

The combination of chlorhexidine with minocycline, doxycycline, meropenem, or ciprofloxacin showed synergistic responses in all clinical isolates, and more than 50% of isolates showed FICI ≤0.5. However, chlorhexidine together with imipenem or levofloxacin showed indifferent responses in 10% and 3.33% clinical isolates, respectively. In all tests, combinations of chlorhexidine with each of the above six antibiotics showed synergistic and additive effects, and inhibited the clinical isolates.

**Conclusions:**

We concluded that, chlorhexidine combined with antibiotics could be used to control the risk of infection with *A. baumannii*.

## Introduction

Chlorhexidine is a bisbiguanide antiseptic, disinfectant and preservative that is effective against a wide range of bacteria, and also a broad-spectrum antimicrobial agent that is active against aerobic and anaerobic bacteria [1, 2]. It is widely used in children and adults with an excellent record of safety and efficacy for applications as diverse as hand washing, preoperative skin preparation, vaginal antisepsis, treatment of gingivitis, and body washes to prevent neonatal sepsis [3–5]. Chlorhexidine acts primarily on the bacterial cell membrane causing leakage of intracellular material. Low concentrations of chlorhexidine affect membrane integrity, whereas high concentrations cause congealing of the cytoplasm [2]. One research has shown that chlorhexidine can decrease the mortality and infection rate in the hospital setting, especially in the intensive care unit [3]. However, other researches have also found that several bacteria, including *A. baumannii, Klebsiella pneumoniae, Pseudomonas aeruginosa, Escherichia coli*, have reduced susceptibility or are resistant to chlorhexidine [6–8].

*A. baumannii* is an opportunistic nosocomial pathogen which can survive for prolonged periods in the hospital environment. *A. baumannii* causes infections including bacteremia, pneumonia, meningitis, septicemia, urinary tract infections, wound and skin infections [9, 10]. The multidrug-resistant (MDR) and extremely-drug resistant (XDR), even the pan-drug resistant (PDR) *A. baumannii* have been found in hospitals. The carbapenem-resistant *A. baumannii w*as identified as a priority by the World Health Organization’s (WHO) report on pathogens requiring research and development of new antibiotics. An important technique to prevent and control the spread of *A. baumannii* infection in the hospital setting is to remove the bacterial cell from the surface of medical devices. In recent years, research has focused on the antimicrobial resistance and reduced biocide susceptibility of *A. baumannii* [10–12]. The chlorhexidine non- susceptibility *A. baumannii* in clinical isolates has been described previously [11]. In addition, chlorhexidine bathing significantly reduces colonization of *A. baumannii* in intensive care unit settings [13]. Özçaka Ö et al [14] found chlorhexidine decreases the risk of ventilator-associated pneumonia in intensive care unit patients, and *A. baumannii* was the most common pathogen (64.7%, 27/34) of all species identified. However, whether bathing can reduce *A. baumannii* infections requires validation with further studies. Therefore, this study aimed to test chlorhexidine combined with antibiotics against the MDR and XDR *A. baumannii* isolated from clinical departments.

## Material and methods

### Bacterial strains and media

*A. baumannii* ATCC19606 was used as the wild-type strain. A total of 30 *A. baumannii* clinical isolates, comprising 6 multidrug-resistant (MDR), and 24 extremely-drug resistant (XDR) isolates, were used in this study and have been described previously [11]. Mueller-Hinton (MH) broth or agar (Oxoid, England) were the growth mediums used throughout the study. Bacteria were cultivated at 37 °C.

### Antibiotics and other agents

The antibiotics and biocides used in this study were purchased commercially. Chlorhexidine acetate, Doxycycline (DOX), minocycline (MIN), levofloxacin (LEV), ciprofloxacin (CIP), imipenem (IMP), meropenem (MER) were purchased from Dalian Meilun Biological Technology (Dalian, China).

### Checkerboard test

The interaction of chlorhexidine acetate with antibiotics was evaluated by the checkerboard method and expressed as the sum of the fractional inhibitory concentration index (FICI) for the DOX, MIN, LEV, CIP, IMP and MER. A 96 well plate was used. Briefly, 170 μl of MH broth medium and 10 μl of bacterial suspension (1.5 × 10^8^ CFU/ml) were added to a 96 well plate and followed by addition of 10 μl a serially diluted chlorhexidine acetate along the x-axis and antibiotics agent on the y-axis. The FICI of each agent was calculated as the minimum inhibitory concentration (MIC) of the agent in combination divided by the MIC of the agent alone. The FICI was calculated in accordance with the following formula:

$$ \mathrm{FICI}={\mathrm{MIC}}_{\mathrm{A}\ \mathrm{combination}}/{\mathrm{MIC}}_{\mathrm{A}\ \mathrm{alone}}+{\mathrm{MIC}}_{\mathrm{B}\ \mathrm{combination}}/{\mathrm{MIC}}_{\mathrm{B}\ \mathrm{alone}}. $$

MIC_A alone_ and MIC_B alone_ are the MICs of drugs A and B when acting alone, MIC_A combination_ and MIC_B combination_ are the concentration of drug A and B in the effective combinations. In the equation, A is chlorhexidine, B is antibiotics agents. The results were interpreted as follows: 0.5 ≤ FICI < 1, synergistic; FICI = 1, additive; 1 < FICI ≤4, indifferent; and FICI > 4, antagonistic [15, 16].

## Results

### Checkerboard test

The checkerboard test showed the synergistic effects of biocides combined with antibiotics against the MDR and XDR *A. baumannii* (Table 1). Meanwhile, the MIC value of antibiotics was one to sixteen-fold decrease which combined with chlorhexidine than alone for clinical isolates of *A. baumannii* (Table 2). The combinations of chlorhexidine with DOX, MIN, MER, or CIP had synergistic effects in all test isolates, as more than 50% of isolates showed the FICI ≤0.5. When chlorhexidine was combined with LEX and IMP, more than 66.67% of isolates were 0.5 < FICI ≤1. However, chlorhexidine together with IMP or LEV were indifferent in only 10 and 3.33% of isolates, respectively. In all tests, chlorhexidine and the other 6 antibiotics showed synergistic and additive effects.

**Table 1:** Effects of biocide combinations on MDR and XDR *A. baumannii* isolates (*n* = 30)

	Antibiotics	FICI range		Mechanism			
		MDR	XDR	Synergistic	Additive	Indifferent	Antagonistic
chlorhexidine	DOX	0.375–1	0.375–1	27 (90)	3 (10)	/	/
	MIN	0.375–1	0.3125–1	27 (90)	3 (10)	/	/
	IMP	0.625–1.5	0.3125–1.5	13 (43.33)	14 (46.67)	3 (10)	/
	MER	0.25–0.625	0.3125–0.75	29 (96.67)	1 (3.33)	/	/
	LEV	0.375–0.75	0.5–1.5	26 (86.67)	3 (10)	1 (3.33)	/
	CIP	0.5–1	0.5–1	26 (86.67)	4 (13.33)	/	/

**Table 2:** The MIC values (μg/mL) of antibiotics which test alone or combined with chlorhexidine for 6 MDR and 24 XDR clinical isolates of *A. baumannii*

Isolates	Country	Age	Source	IMP		MER		LEV		CIP		DOX		MIN		CHG
				MIC _alone_	MIC _combination_	MIC _alone_	MIC _combination_	MIC _alone_	MIC _combination_	MIC _alone_	MIC _combination_	MIC _alone_	MIC _combination_	MIC _alone_	MIC _combination_	MIC _alone_
MDR1	China	48	Sputum	16	16	8	1	4	1	64	16	64	32	16	4	64
MDR2	China	87	Sputum	64	32	64	8	4	2	128	32	64	8	8	1	64
MDR3	China	77	Sputum	16	4	16	4	4	2	64	16	64	16	8	4	32
MDR4	China	72	Sputum	16	4	8	1	8	1	64	16	64	32	16	4	32
MDR5	China	79	Sputum	16	8	4	0.5	8	4	64	32	64	32	16	8	16
MDR6	China	89	Sputum	4	0.5	2	1	8	4	64	32	64	32	8	4	16
XDR1	China	37	Sputum	16	4	8	1	4	1	64	16	32	2	4	1	64
XDR2	China	78	Sputum	64	32	32	8	4	2	128	32	1	0.5	1	0.5	64
XDR3	China	57	Sputum	16	16	8	1	8	4	128	32	64	16	16	4	64
XDR4	China	51	Sputum	64	64	32	2	8	4	128	32	64	16	16	4	128
XDR5	China	78	Sputum	16	1	8	1	8	2	128	32	64	16	16	4	128
XDR6	China	72	Sputum	16	2	8	1	8	4	64	16	64	16	16	1	128
XDR7	China	78	Sputum	64	32	32	4	8	4	128	64	64	16	8	2	64
XDR8	China	74	Sputum	64	32	32	8	8	2	128	32	64	16	8	2	64
XDR9	China	78	Sputum	64	32	64	16	8	2	64	16	64	16	16	2	128
XDR10	China	52	Sputum	64	32	32	2	8	4	128	32	64	16	16	4	64
XDR11	China	61	Sputum	32	16	16	2	8	4	128	64	64	16	8	2	64
XDR12	China	52	Sputum	64	4	32	8	8	2	64	16	64	16	8	2	64
XDR13	China	61	Sputum	32	4	16	4	8	2	128	32	64	16	8	2	64
XDR14	China	93	Sputum	64	8	16	4	8	2	64	16	64	16	8	1	64
XDR15	China	52	Sputum	64	32	16	2	8	2	128	16	64	16	16	2	64
XDR16	China	45	Sputum	16	4	16	2	8	4	128	32	64	16	16	1	64
XDR17	China	82	Sputum	16	4	8	4	8	1	64	16	64	32	8	4	16
XDR18	China	93	Lavage fluid	16	8	8	4	8	8	64	32	64	32	8	4	16
XDR19	China	66	Sputum	16	8	4	1	8	4	64	32	64	32	16	8	16
XDR20	China	80	Sputum	16	8	16	4	8	2	128	64	64	32	16	2	64
XDR21	China	72	Sputum	64	32	16	2	8	2	128	32	64	32	8	2	8
XDR22	China	70	Sputum	16	4	8	1	8	4	64	32	64	32	16	4	32
XDR23	China	66	Sputum	16	8	8	2	8	4	64	16	64	32	16	4	16
XDR24	China	82	Sputum	8	4	8	1	8	4	64	16	64	32	16	2	8
Fold change				1–16		1–16		1–8		2–4		2–16		2–16		

## Discussion

*A. baumannii* has become a major threat in the hospital environment by causing nosocomial infections and colonization. It is inherently resistant to multiple antibiotics [10, 17]. In the present study, MDR, XDR, PDR and biocides non-susceptible *A. baumannii* were isolated from clinical patients [11]. *A. baumannii* clinical isolates have been isolated from sputum samples which frequently lead to pneumonia. In a previous study, the sputum samples accounted for 89.36% of the total samples [11]. Chlorhexidine has good bactericidal effect and high safety on many bacteria. Some clinical research found chlorhexidine decreases the risk of pneumonia by maintaining good oral hygiene and body washing [14, 18] . A recent study has shown that chlorhexidine could reduce the susceptibility to *A. baumannii* infection [11].

Other studies have also shown that chlorhexidine alone or in combination with other antibiotics can prevent and reduce hospital-acquired infections, including respiratory catheter, gastric tube and urinary catheter [13, 19–21]. For example, Jamal et al. [21] showed chlorhexidine alone, or combine with minocycline and rifampicin, reduced the incidence of *A. baumannii* catheter-related infections. In this study, chlorhexidine combinations with doxycycline, minocycline, levofloxacin, ciprofloxacin, imipenem, or meropenem against 6 MDR and 24 XDR *A. baumannii* clinical isolates were tested in vitro. The results showed chlorhexidine combinations with DOX, MIN, MER, or CIP were synergistic in all biocide combinations, with more than 50% of isolates showing FICI ≤0.5. Additionally, the chlorhexidine and antimicrobial MIC values were decreased, which indicates that this technique could effectively reduce drug resistance. However, methods for implementation in clinical settings which avoid adverse drug reactions need to be elucidated. The biocides combined with antibiotics can be used in clinical isolate inhabitation. Since chlorhexidine can be used on the body surface, it can be mixed with antibacterial drugs for disinfectant wipes used in patients’ oral care, tracheal intubation and ventilators, to eliminate fixed-value bacteria and prevent the spread of infection.

The solution of IMP in normal saline solution was stable 3 to 4 h stored at 25 °C [22]. MER in 0.9% saline was stable at least 7 h if the temperature does not increase to 22 °C, while MER stability at 5 h when temperature does not exceed 33 °C [23]. Chlorhexidine combination with MER shown synergistic effects than IMP, and chlorhexidine together with IMP was indifferent in 10% of isolates. Cause IMP easy to lost activity when under test temperature. Meanwhile, solution of CIP was diluted in 0.9% sodium chloride, and the solution was stale at least 3 months when stored at room temperatures [24]. LEV was stable in 0.9% sodium chloride when stored at 25 °C for 3 days [25]. The MIN diluted in Mueller-Hinton agar plate was instable when stored under refrigeration, and DOX also lost activity at a slower rate [26]. The LEV, CIP, DOX and MIN are stable when under test temperature. The combinations of chlorhexidine with DOX and MIN as well as LEV and CIP had the same effects in all test isolates.

## Conclusions

In conclusion, Chlorhexidine combined with antibacterial drugs has synergistic or additive antibacterial effects against multiple and pan-resistant *A. baumannii*. This new technique may have important implications for the treatment and transmission control of *A. baumannii* infection.

## Data Availability

The datasets used and/or analysed during the current study are available from the corresponding author on reasonable request.
